# Postgraduate surgical hernia training with a standardized hernia surgery training tool: training while serving humanity

**DOI:** 10.1097/MS9.0000000000004183

**Published:** 2025-10-28

**Authors:** Basimbe Francis, Isaac Mubezi, Ignatius Kakande, Chris Oppong, Jacob Akoh, Benjamin Desmond Tatumwa

**Affiliations:** a Head of Department Surgery, Head of Gastrointestinal Surgery and Endoscopy St. Francis Hospital Nsambya, Kampala, Uganda; b Mother Kevin Postgraduate Medical School, Uganda Martyrs University, Nkozi, Kampala, Uganda; c Head Department of Surgery, Hope & Healing Center, Kiwanyi, Iganga, Jinja, Uganda; d University of Plymouth, UK

## Introduction

The hernia surgery skills training course was first organized in 2016, after approval by the COSECSA (College of Surgeons of East, Central and Southern Africa) Executive at a meeting in Geneva, through representation by Prof. Laston Chikoya, then Chairman of the COSECSA Educational Committee. Uganda was selected as the hub of the training program, and Prof. Ignatius Kakande was charged with the responsibility of organizing it. He has successfully supervised the collaboration with Operation Hernia in organizing this course.

The Operation Hernia UK-COSECSA Training Course is a comprehensive program in groin Hernia surgery and has had over six previous courses, providing residents with an invaluable training opportunity. It is a structured program with integrated competence assessment, delivered on a one-to-one basis. This model, designed by Operation Hernia and Surgeons for Africa, has also been used to train residents in Rwanda. It has been published and can be applied to other surgical specialties^[1]^.

This study elaborates on a hernia surgery skills training program conducted with surgical residents and COSECSA fellow candidates. The training aimed to achieve an appropriate level of competence in mesh and tissue hernia surgery; train residents who complete the course to become trainers, thereby sustaining the program; and expand the program to surgical residents from other COSECSA countries using the Ugandan experience as a model.

The rationale of this training follows the background of the high burden of hernias, where the prevalence of hernias in African countries is high^[2,3]^. And yet the rate of repair of hernias is low (10–100/100 000) compared to rates in high-income countries^[4]^. This gap highlights a critical need for enhanced surgical capacity and training in the region. Surgical trainees in African countries do not all have access to didactic structured training with competence assessment^[1]^. Addressing this training deficit is essential to improve surgical outcomes and meet the demand for hernia repairs across these populations.

**HIGHLIGHTS**

- Standardized training improved residents’ hernia surgery skills and confidence.
- Mesh repair and tissue repair were taught using a structured, five-stage surgical training model.
- 92.7% satisfaction with lectures, 95.2% with hands-on surgical training sessions.
- Model adaptable to COSECSA (College of Surgeons of East, Central and Southern Africa) countries to address Africa’s hernia surgery gap.
- Mentorship and assessment ensured sustained competence in hernia surgery.

This training program represents a successful partnership between COSECSA and Operation Hernia. To date, the program has trained 56 residents, despite the 4-year gap between 2019 and 2023 due to the COVID pandemic and the Ebola outbreak 2022. The training continues to receive enthusiastic and highly positive feedback from participating surgical residents.

## Methods

Didactic Lectures covering relevant aspects of hernia surgery are delivered at the beginning of the course. These include training videos on the Lichtenstein operation and the Shouldice operation. The videos were produced by Incision Academy^[5]^, a Dutch company specializing in surgical education by e-learning. Dr. Erik-Jan Vlieger, CEO of Incision Academy, granted permission for the videos to be used during the workshop. The second part of the program is a hands-on, one-to-one training in mesh repair (Lichtenstein) and non-mesh repair (Shouldice). This practical training usually spans 5 days with each trainer assigned a maximum of three trainees.

The lectures are delivered at a postgraduate medical school training institution. The theatre sessions take place at a modern medical facility in Eastern Uganda, located in a rural area, which provides excellent facilities for theatre training. A series of evaluations and assessments are integrated into this training program. A pre-course surgical experience survey provides essential background information, enabling trainers to tailor training of residents. Pre- and post-course surveys assess residents’ knowledge of hernia surgery to evaluate knowledge acquired from the lectures. Additionally, residents complete evaluations of both the lectures and theatre training, which serve as a monitoring tool to support continuous improvement of the program.

The competence assessment of residents is usually in two parts: (1) continuous assessment throughout the training, used to tailor learning to individual participants, and (2) a formal end-of-training assessment of competence. As part of the final assessment, residents complete a self-assessment of their competence, which is compared with the evaluation by their trainers.

Mentoring is also an integral part of the training. The results of the formal assessments are shared with the residents’ supervisors for mentoring in their hospitals. Some residents benefit from dedicated mentoring sessions organized as part of the training course.

### Hands-on training

This component of the course is delivered as an intense and effective “apprenticeship.” Each trainer reviews the documented surgical experience of their assigned trainees to inform them of their training strategy. Trainers adhered to a structured sequence of steps in instructing their trainees: demonstration of groin anatomy, delivery of spermatic cord, dissection of hernia sac, management of hernia sac, assessment & management of posterior wall, and management of cremaster muscle and insertion of mesh. Trainers also evaluate general surgical skills like tissue handling and appropriate use of surgical instruments. Particular emphasis is placed on identifying all the anatomical structures, including the three main nerves – ilioinguinal, iliohypogastric, and genital branch of the genitofemoral nerves. Correct identification of the nerves reduces risk of injury, ischemia, and entrapment, all of which contribute to chronic groin pain.

**Mesh repair**: The appropriate technique of mesh insertion is demonstrated and taught, highlighting the critical steps that would reduce recurrence as well as other short- and long-term complications.

**Non-mesh repair**: Non-mesh or tissue repair remains an important option in the African setting because of limited availability of mesh and unsuitability of mesh insertion in contaminated hernia wounds. Shouldice repair – rather than Bassini repair – was demonstrated and taught during the course.

**Figure 1.**
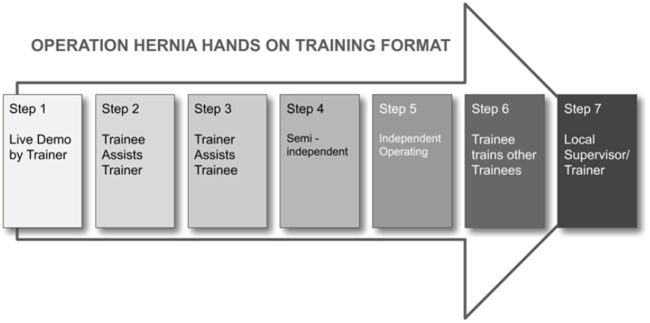
Hands-on training model depicting the format of the adopted training program.

The format of the training adopted is depicted in Fig. 1 and explained in Table 1.

**Table 1:** Detailed explanation of training format

Steps	Details
Step 1	The trainer demonstrates the procedure while the trainee assists.
Step 2	The trainee assists the trainer multiple times depending on the trainee’s prior knowledge, skill, and aptitude, as well as the trainer’s assessment.
Step 3	The trainer assists the trainee in performing parts of the procedure, or the entire operation, depending on the trainee’s skill and learning progress.
Step 4	Semi-independent: The trainee performs the procedure with assistance from a fellow trainee, under supervision of a trainer who may be scrubbed or unscrubbed, but is prepared to scrub in, provide instruction, and assist as needed.
Step 5	Independent operating: The trainee performs the procedure independently, with the trainer supervising from a distance.
Step 6	The trainee trains other trainees.
Step 7	Local supervisor/audit: The trainee is assigned a local supervisor who provides opportunities to maintain the newly acquired skills and audit their logbook.

The entire hernia operation is deconstructed into several steps, with each “stage” in the Training Format comprising several components of the deconstruction.

Progression from one stage to the next depends on satisfactory assessment by the trainer. At each stage, the trainee engages in a debrief session with the trainer to reflect on their performance and assess their competence.

### Precourse survey of surgical experience

In order to assess residents’ general surgical experience and their level of exposure to hernia surgery, a pre-course self-assessment survey is administered. This provides valuable background information facilitating trainer allocation and supports tailored training for each resident.

## Results

Over the past 8 years, 56 residents have been trained in open hernia surgical skills. A total of 23 trainers were in the program during this period, as shown in Table 2.

**Table 2:** Number of residents and trainers in the hernia surgery workshops over the years

Year	Number of residents	Number of trainers
2016	10	5
2017	9	3
2018	9	4
2019	12	4
2023	7	3
2024	9	4
Total	56	23

Fifty-six residents have been trained in open hernia surgical skills in the last 8 years.

### Knowledge improvement in hernia surgery

**Figure 2.**
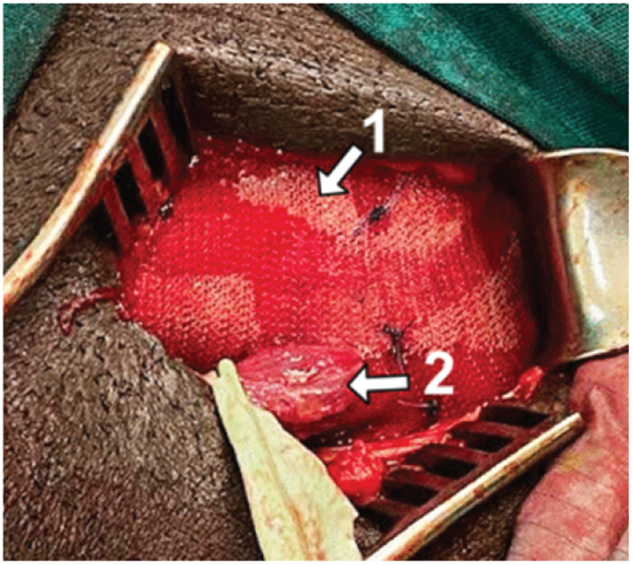
Mesh repair performed by a resident. *Arrow 1* indicates mesh insertion, and *Arrow 2* indicates the spermatic cord.

Improvement in the overall knowledge of hernia surgery was measured using a score from pre- and post-course self-assessment feedback forms completed by the residents. Improvements were also observed across other aspects of hernia surgery discussed during the course (Figure 2).

### Residents’ feedback

This component is a crucial part of the training program, as effective feedback from residents informs the development and improvement. Trainees provided a formal, independent, and anonymized assessment of the training workshop by completing a feedback form. They evaluated all aspects of the course and included their general comments. Feedback on both lectures and the hands-on training was extremely positive.

### Feedback: lectures

**Figure 3.**
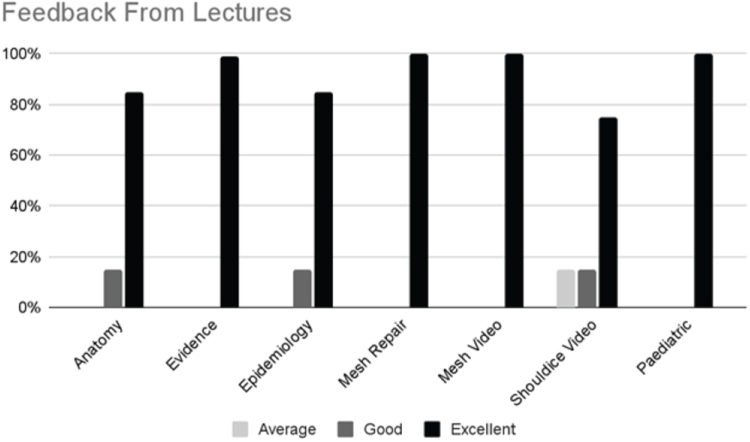
Feedback on lectures with three color categories representing average, good, and excellent across different subjects, anatomy, evidence, epidemiology, mesh repair, mesh video, Shouldice video, and pediatric surgery.

1. 92.7% of responses regarding the lectures were “good” to “excellent.”
2. 64% were “excellent” (Figure 3).

### Feedback: theatre training

**Figure 4.**
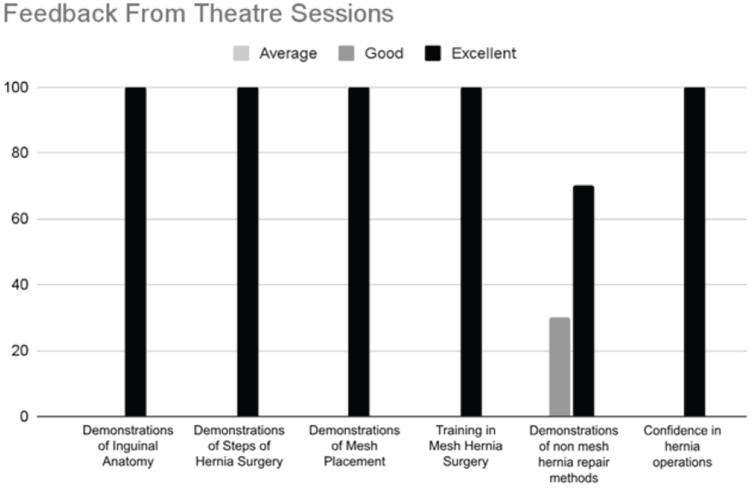
Feedback from theatre sessions covering demonstration of steps of hernia surgery, demonstration of mesh placement training in mesh hernia surgery, demonstration of non-mesh hernia repair methods, and confidence in performing hernia operations.

Of the responses, 95.2% regarding the theatre sessions were “good” to “excellent.” More time will be allocated to non-mesh repair in subsequent hernia courses (Figure 4).

This very high approval rating from the residents is evidence of the quality of the training program they received and the high level of engagement from trainers.

### Feedback comments from residents

A sample of trainee comments that stand out are listed below:

1. All residents have confirmed that the hernia course has improved their surgical skills and confidence in hernia surgery.
2. Feedback and critique from trainers were good.
3. Initially, I was very bothered about hernia operation because I never had one-to-one training and exposure. Today after the camp, I can say that I am very confident about the operation.
4. I had never seen a nerve in a hernia operation. I can now identify the relevant nerves.
5. I can properly identify all the relevant structures. I can confidently insert the mesh.
6. I dealt with fear of dissecting the sac.
7. Learned to dissect the sac bloodlessly along the correct planes.
8. I learned how to infiltrate local anesthesia.
9. I have never been confident that I can handle all types of hernia operations like I felt at the end of this training. With the knowledge I have acquired and the recognition of relevant anatomical structures, I feel I can find my way into safely operating on a hernia patient despite the variations.

## Conclusion

The course has been an overwhelming success, judging by the assessment of especially the residents but also by the organizers on behalf of COSECSA and the Operation Hernia UK surgeons”

I have never been confident that I can handle all types of hernia operations, like I felt at the end of this training. With the knowledge I have acquired and the recognition of relevant anatomical structures, I feel I can find my way into safely operating on a hernia patient despite the variations.

This feedback from a resident sums up the success of the training program.

It is imperative, therefore, that more surgical trainees are given access to this essential training opportunity.

## Data Availability

The datasets generated and/or analyzed during the current study are available from the corresponding author on reasonable request.

## Research registration unique identifying number (UIN)

Name: Postgraduate Surgical Hernia Training with a Standardized Hernia Surgery Training Tool: Training While Serving Humanity.

UIN: researchregistry11177.

Hyperlink: https://researchregistry.knack.com/researchregistry#home/registrationdetails/680b38290141a402abbdf8b8/.
